# The Potential Role of Metabolomics in Drug-Induced Liver Injury (DILI) Assessment

**DOI:** 10.3390/metabo12060564

**Published:** 2022-06-19

**Authors:** Marta Moreno-Torres, Guillermo Quintás, José V. Castell

**Affiliations:** 1 Unidad de Hepatología Experimental, Instituto de Investigación Sanitaria Hospital La Fe, 46026 Valencia, Spain; 2 CIBEREHD, Instituto de Salud Carlos III, 28029 Madrid, Spain; 3 Unidad Analítica, Instituto de Investigación Sanitaria Hospital La Fe, 46026 Valencia, Spain; gquintas@leitat.org; 4 Health and Biomedicine, LEITAT Technological Center, 46026 Valencia, Spain; 5 Departamento de Bioquímica y Biología Molecular, Facultad de Medicina, Universidad de Valencia, 46010 Valencia, Spain

**Keywords:** DILI, metabolomics, hepatotoxicity, biomarkers, diagnostic

## Abstract

Drug-induced liver injury (DILI) is one of the most frequent adverse clinical reactions and a relevant cause of morbidity and mortality. Hepatotoxicity is among the major reasons for drug withdrawal during post-market and late development stages, representing a major concern to the pharmaceutical industry. The current biochemical parameters for the detection of DILI are based on enzymes (alanine aminotransferase (ALT), aspartate aminotransferase (AST), gamma-glutamyl transpeptidase (GGT), alkaline phosphatase (ALP)) and bilirubin serum levels that are not specific of DILI and therefore there is an increasing interest on novel, specific, DILI biomarkers discovery. Metabolomics has emerged as a tool with a great potential for biomarker discovery, especially in disease diagnosis, and assessment of drug toxicity or efficacy. This review summarizes the multistep approaches in DILI biomarker research and discovery based on metabolomics and the principal outcomes from the research performed in this field. For that purpose, we have reviewed the recent scientific literature from PubMed, Web of Science, EMBASE, and PubTator using the terms “metabolomics”, “DILI”, and “humans”. Despite the undoubted contribution of metabolomics to our understanding of the underlying mechanisms of DILI and the identification of promising novel metabolite biomarkers, there are still some inconsistencies and limitations that hinder the translation of these research findings into general clinical practice, probably due to the variability of the methods used as well to the different mechanisms elicited by the DILI causing agent.

## 1. Introduction

### 1.1. DILI and Its Clinical and Pharmaceutical Relevance

DILI is defined as a liver injury caused by pharmaceuticals, herbs, or other xenobiotics, resulting in abnormalities in liver tests or liver dysfunction [1]. DILI due to prescription or over-the-counter drugs is the most common manifestation of drug hepatotoxicity and a growing public health issue [2]. It represents the major reason for liver transplantation and is the leading cause of acute liver failure (ALF) in Europe and the United States. DILI accounts for 7–15% of the ALF cases (acetaminophen overdose excluded). Acetaminophen itself represents over 50% of ALF in adults in the USA, being the drug most frequently involved [3,4,5,6]. Unexpected adverse hepatic reactions to clinical drug treatments have an estimated incidence varying from 2.4 to 19 per 100,000 patients per year [7,8,9]. Its incidence and morbidity are rising in parallel to the introduction of new drugs, the extension of life expectancy, poly-medication in elderly people, and the widespread use of over-the-counter “alternative” medicines including herbal products.

On the other hand, DILI has also a significant relevance in the pharmaceutical industry business. Despite the rigorous steps in the early phases of drug development including the preclinical phase and the clinical trials [10], it is impossible to have complete information and 100% certainty about the safety of a new drug at the time of its approval. Thus, DILI is one of the most frequent and unexpected reasons for drug development discontinuation, drug non-approval, post-marketing regulatory additional actions, and withdrawal from the market [2]. Hence, the assessment and early recognition of hepatotoxicity of new drugs are of great interest to the pharmaceutical industry.

### 1.2. Types of DILI and Clinical Features of DILI

There are drugs (intrinsic hepatotoxins) that cause hepatocellular damage by defined mechanisms in a predictable dose-dependent manner and in all individuals exposed. This makes the identification of such compounds easier at the early stages of drug development. Others (idiosyncratic hepatotoxins) cause damage for reasons that are closer to the individual phenotypes rather than to the drug itself, and hence, the event is rare, unpredictable, and not always clearly related to dose, route, or treatment duration. Idiosyncratic hepatotoxicity can be classified into metabolic and immunological categories. In the first case, DILI susceptibility depends on a particular patient’s environmental setting (concomitant disease), individual’s genetic susceptibility, host-related (age, gender, and ethnicity), and drug-related factors (dose, metabolism, and lipophilicity) [11,12]. In the second case, the major player is the activation of the immune system recognizing drug-derived antigens on hepatocytes and triggering a hypersensitivity Type I or IV reaction that ultimately can evolve into an autoimmune disease [12]. Typically, idiosyncratic toxicity occurs with drugs and at doses otherwise well tolerated by the majority of patients [13].

Three patterns of damage are the hallmark for the classification of DILI, namely, hepatocellular, cholestatic, and mixed type, as adopted by the Council for International Organisations of Medical Sciences [14,15]. Hepatocellular DILI is characterised by a liver cell necrosis pattern, release of transaminases ALT and AST and lactate dehydrogenase (LDH), and inflammation, but only mild bile stasis [11]. This hepatocellular pattern is present in acute hepatic necrosis, acute hepatitis, chronic hepatitis, and non-alcoholic steatohepatitis (NASH) [16]. On the other hand, the cholestatic DILI phenotype is characterised by bile production impairment and reflux with bile stasis, which affects ductal cells and the biliary tree with an increase in ALP and GGT in sera. Because of bile flux stasis, other conjugates normally excreted to bile are also increased in serum, (i.e., conjugated bilirubin). The mixed-type DILI shares features of both disease phenotypes and is typical of many drugs and the most frequent pattern of DILI, otherwise rarely occurring in other forms of acute liver disease.

### 1.3. The Diagnosis of DILI: Conventional Clinical and Biochemical Biomarkers

Currently, there are no specific biomarkers for the early and conclusive diagnosis and monitoring of DILI. Thus, the pathological examination of a liver biopsy remains the gold standard providing the most conclusive insight into the pathophysiological nature of a drug-induced liver damage event. Yet, it is not free from risks and side effects for the patient and therefore is not always applicable. In practical terms, DILI is diagnosed by excluding other putative liver diseases, and by the application of causality scales that assign a probability degree to an event as being caused by a drug [17]. The temporal relationship between drug intake and the clinical symptoms is determinant in the score provided by causality scales. However, reactions may occur from weeks to months after treatment initiation or even appear once the administration of the drug has been discontinued [18,19]. The situation becomes even more complex in multi- or poly-medicated patients and/or with concomitant comorbidities. In many cases, re-challenging with the drug, whenever acceptable, renders them unique and definitive proof to identify the drug responsible for DILI [20].

To assist in the classification of the different DILI phenotypes, serum enzymes AST, ALT, ALP, GGT and total bilirubin (TB) can be used. ALT levels are more liver-specific than AST, but are not aetiology specific [21,22,23]. ALP is also not liver-specific and can be considerably elevated in other liver non-related diseases [24]. Regarding bilirubin levels, they only increase when there has been extensive liver damage. Moreover, jaundice runs parallel to cholestasis in most but not in all DILI cases.

In this regard, Hy’s law is an empirical estimation of the severity and risk of suffering an ALF as a consequence of a DILI episode. It states that drug-induced jaundice together with hepatocellular injury, without a significant obstructive component, is an empirical indicator of the probability of suffering ALF. It is based on the evidence that jaundice as expressed by TB in blood, decreased hepatic function, expressed by the International Normalised Ratio of prothrombin time test (INR), and a high ratio of AST or ALT, can predict the risk of developing ALF. ALF is very likely to occur when hepatic injury (ALT/AST > 3 × upper limit of normal (ULN)), jaundice (TB > 2 × ULN), or liver dysfunction (INR > 1.5 × ULN) and in the absence of obstructive cholestasis (ALP < 2 × ULN) and excluded from other disease-induced liver damage [25].

Based on the ratio of ALT and ALP enzymes and their value over the upper normal levels, clinicians easily classify the various phenotypes of DILI using an R-value defined as [ALT value/ALT upper normal limit (UNL)]/[ALP value/ALP UNL]). DILI is assigned as hepatocellular when R ≥ 5, cholestatic when R ≤ 2, and mixed type if 2 < R < 5 [26]. The R index may display the same value, independently of the absolute magnitudes of ALT and ALP as far as their relative ratio remains constant and does not assist in better estimating the nature of the “mixed-type” DILI phenotype. In general, in the course of DILI, levels of serum liver enzymes do not correlate with the degree of hepatocyte metabolic dysfunction, histological patterns of damage, or severity [26], rather they increase only upon substantial and massive hepatocyte damage [27]. In addition, mitochondrial respiratory chain inhibition, or mitochondrial dysfunction that often occurs at the early stages of drug-induced injury is not accompanied by elevated ALT or ALP values [28], preventing an early diagnosis of DILI. These drawbacks limit the confident use of the R-score for a precise phenotype diagnosis and progression of DILI.

Several genetic and circulating biomarkers for the estimation of the risk of suffering a DILI event have been proposed by the large-scale initiative of the Safer and Faster Evidence-based Translation (SAFE-T) Consortium in Europe. These include glutamate dehydrogenase (GLDH), glutathione S-transferase, high-mobility group box-1 (HMGB-1), miRNA-122, full length, and caspase-cleaved keratin-18 (K-18) [29,30,31], different HLA alleles [32,33,34], and sorbitol dehydrogenase (SDH). However, altered levels of these biomarkers are not specific to DILI or liver disease [35,36].

## 2. Metabolomics in DILI Research and Diagnosis

### 2.1. Metabolomics vs. Transcriptomics and Proteomics in DILI Biomarker Discovery

The use of *omics* techniques for the identification of the biochemical changes caused by hepatotoxic drugs, and hence, uncovering new DILI biomarkers is an active field of research. In the past decade, transcriptomics and proteomics have been widely used for the study of hepatotoxicity [37,38]. These techniques help to visualise effects of hepatotoxins on gene activation and protein expression, but cannot reveal subtle changes in the cell’s metabolism, the end step of any disturbing action elicited by a toxicant. The liver plays a key role in the homeostasis of the whole organism, with many active metabolic processes susceptible to being altered by the noxious effects of a drug. Thus, it is reasonable to assume that the effects of a drug ultimately causing DILI are reflected in changes in the metabolome [28,39]. While genomics and proteomics describe what might be potentially happening, given that metabolites are the end products of the metabolic reaction pathways, metabolite alterations, and the metabolome provide the best comprehensive insight into what is actually happening as a consequence of a noxious stimulus [40]. They better represent the actual metabolic status of a cell or tissue being a relevant source of information to identify the molecular initiating events in the context of the *Adverse Outcome Pathway* (AOP) approach [41]. Metabolites are commonly present in many cell types, tissues, and organisms and are part of many conserved metabolic pathways; thus, the analysis and translation of results to humans are affordable [42].

Metabolomics also represents an interesting approach regarding toxicity response prediction, based on the identification of preclinical indicators of susceptibility to hepatotoxins that can be anticipated by assessing the basal pattern of endogenous metabolites prior to drug administration [43,44]. Indeed, several studies have evidenced that humans display characteristic and individual metabolic phenotypes or “metabotypes” [45,46,47] which are intended to be used to anticipate the distinct responses to drugs in drug metabolism, and therefore susceptibility to DILI, advocating for individualized drug therapies [44,45,46,47,48].

### 2.2. Metabolomics for the Study of DILI Pathophysiology

**Figure 1:**
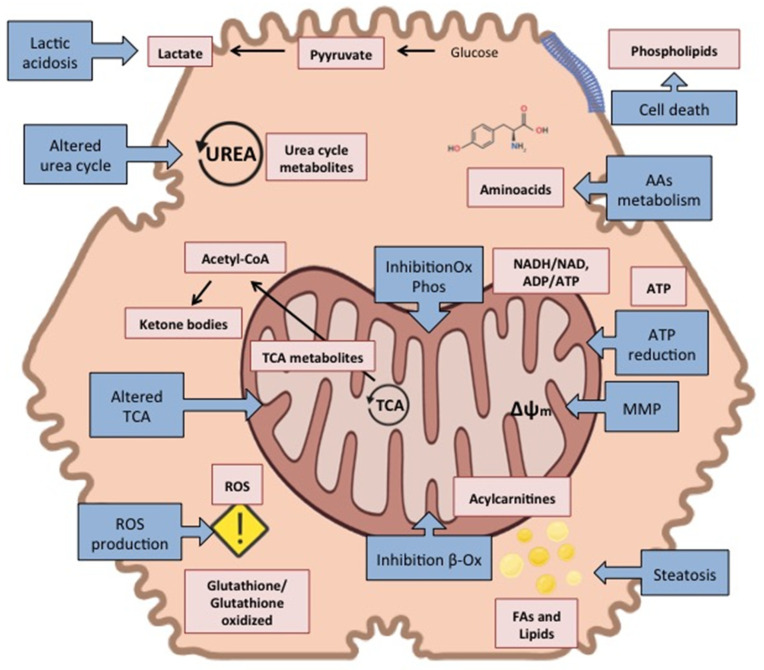
Schematic representation of the principal biochemical mechanisms of drug-induced hepatocellular damage and the metabolite alterations involved.

The onset of DILI may occur via different molecular mechanisms. Active hepatotoxins act *per se*, interfering enzymes, receptors, ion channels, or critical biomolecules, thus causing cell metabolism interferences. For latent hepatotoxins, the metabolites generated in the course of Phase I biotransformation reactions result in the bioactivation of the parent compound generating a more reactive/toxic metabolite that ultimately causes the harm. Parent drugs or their reactive metabolites are usually inactivated by Phase II metabolism. However, when conjugation pathways are overwhelmed, the parent drug, the drug-derived metabolites, drug-derived reactive species/radicals, or reactive oxygen species generated in the course of these reactions, can exert cellular stress by a wide range of mechanisms (Figure 1). In this context, metabolomics allows the detection of the altered cell’s metabolism in the course of a DILI event. Mitochondria frequently stay in the center of many different DILI injury events. Drugs can also impair the mitochondrial respiratory chain, induce mitochondrial membrane permeability transition (MPT) and decrease oxidative phosphorylation, which results in adenosine triphosphate (ATP) depletion, and the generation of excessive reactive oxygen species. Impaired levels of ATP, adenosine monophosphate (AMP), reduced nicotinamide adenine dinucleotide (NADH), oxidized nicotinamide adenine dinucleotide (NAD+), glutathione and oxidised glutathione can be measured as metabolic readout. Direct mitochondrial injury can also impair ß-oxidation of fatty acids which results in altered lipid metabolism and lipid accumulation (steatosis), as well as impacts on the acylcarnitine levels. Additional effects of mitochondrial impairment are the accumulation of ketone bodies as a consequence of the inability to oxidise Acetyl-CoA in the tricarboxylic acid cycle (TCA) together with altered levels of TCA metabolites.

Altogether, MPT activation and subsequent release of cytochrome c is a common step that further mediates cell death by apoptotic or necrotic mechanisms. As a consequence of these cell-death-related events and given the central role of hepatocytes in amino acid metabolism, an increase in proteolysis, as well as improper amino acid metabolism, may lead to increased amino acid levels. Additionally, an increase in phospholipid levels might be observed in relation to cell death processes since they are principal components of the cellular membrane.

Furthermore, the block in the flow of electrons in the respiratory chain because of the impaired mitochondrial respiration, decreases the reoxidation of NADH into NAD+, decreasing oxidation of pyruvate by the pyruvate dehydrogenase which is then reduced into lactate triggering lactic acidosis.

As hepatocytes account for a large number of metabolic functions to preserve organism homeostasis, drugs can affect many of them, such as increasing glycolysis with an associated lactic acidemia, impairing gluconeogenesis, the ammonia conversion into urea, and amino acid interconversions, all of them having a strong influence on hepatocyte’s metabolome.

There is also a significant involvement of the urea cycle likely related to mitochondrial dysfunction, hepatocyte death, and the hepatic adaptation pathway, where metabolomics also plays a significant role in measuring the impact on the levels of the metabolites involved.

**Figure 2:**
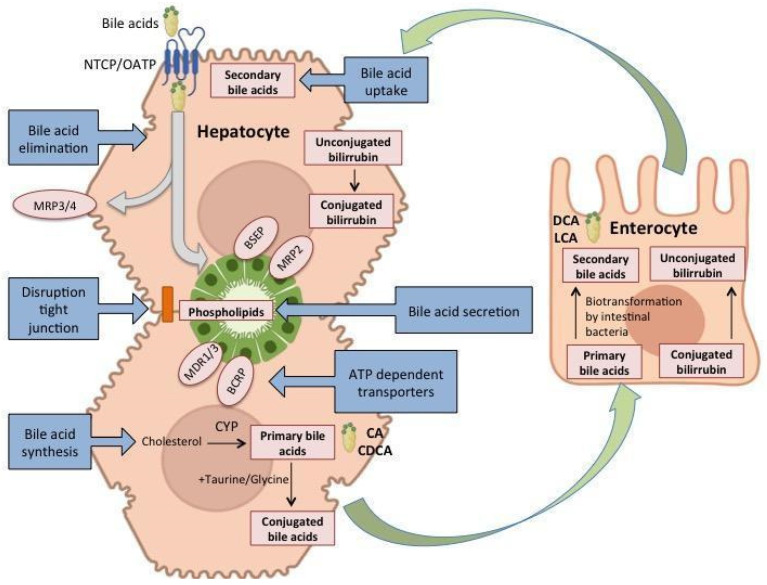
Schematic representation of the biochemical mechanisms in drug-induced cholestasis. Primary bile acids cholic and chenodeoxycholic acid are synthesized by hepatocytes, conjugated with taurine, glycine, and sulphate, and excreted into bile, reaching the intestine. There, the bacterial flora modifies them, de-conjugating and oxidizing to deoxycholic and lithocholic acid which is reabsorbed into blood, uptake by hepatocytes and conjugated again. There are ca. 40 different bile acid species in humans that can be properly analysed by metabolomics [49] and help to discriminate among the different causes of cholestasis.

Drug-induced hepatotoxicity may result in alterations in the synthesis, conjugation, and secretion of primary bile acids and the uptake of secondary bile acids reabsorbed from the gut into the blood. Hepatotoxins can influence bile acid synthesis as well inhibit cellular transport functions such as the apical (canalicular) bile salt efflux pump (BSEP). This is the major transporter of bile salts and drug metabolites from hepatocytes into bile leading to intracellular accumulation of bile acids that may cause hepatocyte damage. Upon BSEP inhibition, basolateral efflux systems (MRP3 and MRP4) are potential salvage systems to lower the burden of bile salts and drug metabolites for hepatocytes. Drug metabolites are also MRP2 substrates, so this canalicular export system constitutes together with MRP3 and MRP4 potential susceptibility factors for drug-induced cholestasis (Figure 2).

Drug hepatotoxicity is not limited solely to hepatocyte injury. Other hepatic cells may also be affected by drugs, such as endothelial cells resulting in inflammation and sinusoidal occlusive syndrome, or cholangiocytes, resulting in inflammation, destruction and bile flux inhibition, and stasis that affects the excretion of other conjugates, (i.e., bilirubin) [40].

Additional molecular mechanisms involved in drug-induced cholestasis include impaired trafficking and disruption of the tight junction network, destruction of the cytoskeleton, and inhibition of ATP-dependent transporters [50].

Therefore, altered bile acid levels (conjugated and non-conjugated) and other bile components such as phospholipids are four good candidates to be measured by metabolomic approaches as specific cholestatic DILI biomarkers.

Furthermore, hepatic stellate cells (HSCs), which represent approximately 5–8% of the cells in the liver, produce several pro-inflammatory cytokines that may aggravate the drug-induced inflammation. A protein exclusively produced by HSCs, Cytoglobin (CYGB), is involved in acetaminophen (APAP)-induced hepatotoxicity [51]. CYGB deficiency in HSCs can regulate hepatic O2 levels and can alleviate the acute liver injury induced by APAP, by decelerating CYP2E1 bioactivation in hepatocytes [52]. Additionally, HSCs produce several pro-inflammatory cytokines that may aggravate the drug-induced inflammation [53].

An interesting consideration is the fact that among the intracellular metabolites that are altered by the effects of drugs (*endo* metabolome), only those metabolites that are specific to the liver, released from hepatocytes, and are stable enough in the blood, are potentially useful as DILI biomarkers for diagnosis and follow-up purposes. The analysis and relationships between liver *endo*- and *exo*-metabolome provide a high level of information regarding the type and severity of liver toxicity after a chemical insult, but this prerequisite reduces the number of metabolites potentially useful to diagnose and follow up DILI events in vivo.

### 2.3. General Principles for MS-Based Metabolomics Analysis and Biomarker Identification in DILI

**Figure 3:**
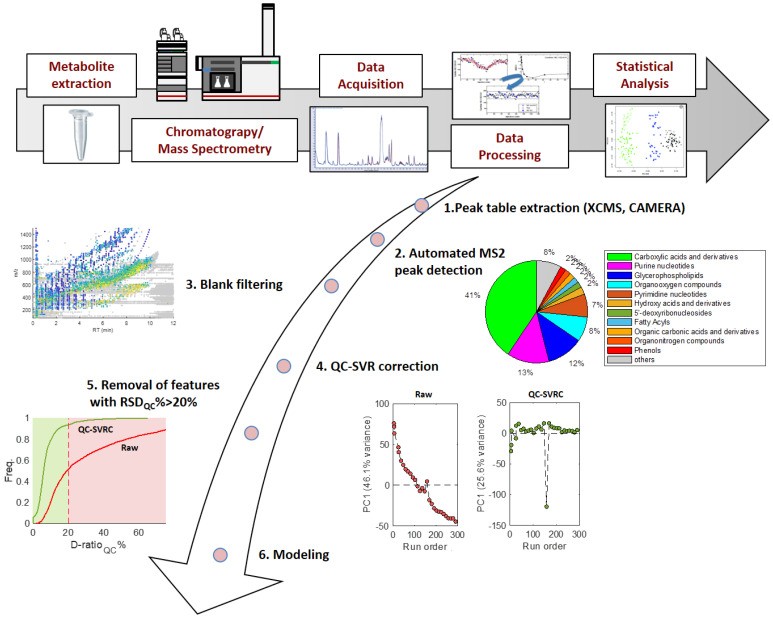
The overall workflow in metabolomic analysis. Robust data acquisition from DILI patients, normalisation and elimination of sources of variability and contamination are key for conclusive bioinformatic analysis that will allow identification of DILI biomarkers.

In any metabolomic analysis, the quality of raw data for further evaluation and interpretation is critical. Sample generation for DILI studies, having in mind they come from patients and are taken at different dates, should be performed carefully and following previously designed and well-established protocols to account for or minimise the impact of external sources of bias and potentially confounding factors. These external sources of bias may distort the true associations between metabolites and injury and modify the relationships among the variables under study [54]. A typical workflow of a metabolomic study for DILI biomarker identification is shown in Figure 3.

Metabolomics can be satisfactorily applied to a wide variety of biological samples. Plasma/serum are frequent clinical samples that are easy to collect and handle [55]. Because of the complexity of the metabolome and the diversity in physico-chemical properties and concentration ranges of metabolites, no single preparation procedure or detection platform allows the quantification or even detection of all the metabolites in a given biological sample. The two analytical platforms mainly used in the metabolomic analysis are nuclear magnetic resonance (NMR) spectroscopy and mass spectrometry (MS). NMR is quantitative and may be used with bulk samples without separation techniques but is limited in the number of metabolites that can be simultaneously analysed, and the equipment is costly and technically demanding [1,56,57,58,59]. On the contrary, MS coupled to ultra-performance separation techniques, such as liquid chromatography (LC-MS) or gas chromatography (GC-MS) is very sensitive, allows simultaneous analysis of hundreds of metabolites, is cost affordable and is currently the most effective and commonly used detection technology as it combines the sensitivity, specificity, and linear range together with the information provided by the chromatographic separation. While GC is required to analyse small, thermally stable, volatile, and easily gasified compounds, LC is best used to analyse polar, higher relative molecular mass, low volatility, and lower thermal stability metabolites [57]. Regarding LC, reverse phase (RP) chromatography is suited for the analysis of medium and low polarity compounds while hydrophobic interaction chromatography (HILIC) is preferred for the analysis of polar and polar ionic compounds, which are quite abundant in biological fluids, (e.g., amino acids, sugars, phospholipid, conjugates) [60]. No matter how data is retrieved, (e.g., type of ionisation or chromatographic separation) they can ultimately be jointly analysed to maximise the metabolic coverage, increase confidence in the identification of potential biomarkers and elucidate the biological events in toxicity [61]. Serum/plasma sample analysis does usually require the combination of both approaches to ensure the broad coverage of metabolite types when searching for new biomarkers.

Following the step of data generation and acquisition, several statistical tools are required to process raw spectra and generate a peak table [62]. There are commercial and freely available software packages for the automation of operations involved, (e.g., peak detection and alignment, integration, and identification of isotopic profiles [63,64,65], such as MetAlign, MZMine, and XCMS [66,67].

A common objective in metabolomic studies of DILI is the identification of metabolic patterns characteristic of toxic responses. The development of algorithms for metabolite annotation is a very active field of research, as well as the creation and enlargement of spectral databases such as Human Metabolome Database (HMDB), METLIN, and ChemDB to facilitate metabolite identification. In spite of that, metabolite annotation is currently the main bottleneck in metabolomics and the percentage of annotated features in metabolomic studies is often low (<10–20% of the detected features) [68,69]. Furthermore, signals from chemicals present in reagents such as mobile phases or mobile phase additives, and contaminants derived from sample collection, handling, and processing are also present in the raw data. To eliminate these uninformative features from the data set, blank samples are usually included in the batch analysis to subtract uninformative signals.

It also happens that gradual systematic changes in the system performance, during the analysis of a batch of samples, electronic drifting, and the effects of ion suppression or enhancement, can compromise the accuracy, repeatability of the methods, and reproducibility of the results from downstream analysis. Therefore, quality control (QC) samples are required to be analysed along the process of data acquisition and are very useful to correct and normalise readings. This improves the reproducibility of mass spectra providing consistent metabolomics data. Thus, samples are analysed in a random order and QC samples are analysed at the beginning and at the end of the batch, as well as periodically distributed throughout the batch, to monitor the system stability and technical performance, and to implement effective batch effect correction afterward [70]. The systematic within-batch effects vary in direction and linearity depending on the type of metabolite, and several algorithms have been developed to approximate the underlying error function [71,72]. After batch effect correction, features showing low repeatability in QC samples can be considered unreliable and removed from further analysis. A widely used estimate is the dispersion ratio, and LC–MS features with a dispersion ratio (D-ratio*) > 20% are often excluded from the data set.

Finally, given the large amount of data generated in metabolomics approaches and the strong influence of key experimental factors, (e.g., processing day, storage time, compound treatment) and instrumental factors (batch effect) [54], bioinformatic tools and statistical analysis are required in order to extract the maximum information, and metabolic pathway analysis to obtain meaningful biological findings [73]. These chemometric analyses are essential to develop pattern recognition models, detect biomarkers from highly dimensional omics datasets and define the context of their changes in the case of DILI, in view of the aetiology and pathogenesis of liver injury.

### 2.4. Metabolites Identified as Putative Biomarkers of DILI in Humans

Considerable advances have recently been made in metabolomics in the discovery of useful biomarkers. Here, we review the major outcomes of metabolomics studies performed for DILI evaluation. A literature search was conducted using the terms “metabolomics”, “DILI” and “humans” in the PUBMED, Web of Science, PubTator, and Embase databases. In vitro and animal studies were excluded from the revision. Despite some initial studies being performed with NMR, MS was the prominent methodology used in the vast majority of research since it provides different ionisation techniques, increasing the number of metabolites that can potentially be detected with enormous sensitivity and selectivity. Being an emerging area of research, the number of scientific reports is reduced. Table S1 (Supplementary Material) summarises metabolic alterations associated with DILI and provides a list of metabolites consistently reported between 2009 and 2021 in 25 clinical and experimental studies, considered as emerging circulating DILI biomarkers. Attention was paid principally to their links to the DILI and not to the drug causing the event. Among them, few studies addressed the identification of specific markers for DILI prediction or individual susceptibility determination [43,47,74,75,76] while the vast majority were focused on DILI diagnosis and follow-up [55,77,78,79,80,81,82,83,84,85,86,87,88,89,90,91,92,93,94,95].

Acetaminophen (APAP) has been the most commonly used model of intrinsic DILI as it is the most clinically relevant [48]. Specific methods of capillary electrophoresis hyphenated to mass spectrometry have been described for the determination of acetaminophen and its main metabolites in urine [53], which have been shown to correlate with hepatic damage after APAP overdose [96]. Two papers included in this review showed downregulation of urinary acetaminophen sulphate and the ratio of acetaminophen sulphate to acetaminophen glucuronide, whereas there is an increase in acetaminophen cysteine [43,47]. In addition, the TCA cycle appeared as a significantly altered pathway upon APAP overdose [84,95], suggesting that hepatic dysfunction or failure might be induced by inadequate function of TCA, which correlates with suppressed oxidative phosphorylation and increased anaerobic glycolysis.

Among the papers reviewed, a subset of metabolites appeared consistently altered in different studies, reinforcing their potential as DILI biomarkers. That was the case of lactate, which appeared to increase in [47,55,79,84,95], in agreement with other studies in rat serum [97], mouse plasma [98], or pig plasma [99]. Lactate is metabolised mainly by the liver, so increased levels might reflect decreased clearance due to impaired hepatic function. It may also be the consequence of exaggerated glycolysis in the liver and can escape from hepatocytes. Bernarl et al. [100] also showed that lactate levels are significantly higher in non-surviving patients than in survivors from APAP-induced acute liver failure. Enhanced levels of lactate and glucose were observed in two additional studies [84,95]. All this is consistent with a mitochondrial impairment that can lead to the inability to oxidise pyruvate by the pyruvate dehydrogenase leading to its reduction to lactate triggering lactic acidosis. Accordingly, an increase in pyruvate and alterations in the citric acid cycle as a consequence of mitochondrial dysfunction are observed in three [74,79,84] and four [84,86,91,95] studies, respectively. A decrease in citrate acid is expected to correlate with suppressed oxidative phosphorylation and increased anaerobic glycolysis. Lowering the oxidative phosphorylation inhibits the production of hippurate in hepatic mitochondria [101,102]. Decreased hippurate concentration is also expected to occur upon hepatic failure. In that sense, hippurate appeared to increase urine secretion in two studies [47,84] while it was reduced in a different one [95]. Further insights have to be accomplished to interpret these discrepancies. The decreases in urinary citrate and hippurate were in accordance with preclinical studies in rats and mice [97,98,103].

Glucose metabolites such as glucuronic acid appeared to increase in two studies [88,90]. Glucuronic acid conjugates with xenobiotics, such as drugs and bilirubin, with a high probability of making them more water-soluble and eliminating them from the body through the urine or bile. The significantly elevated levels found in these studies might induce impairment in liver detoxification.

Furthermore, when acetyl Coenzyme A (CoA) derived from ß-oxidation of fatty acids exceeds the capacity of being incorporated into the Krebs cycle, two molecules of acetyl-CoA condense to form acetoacetyl-CoA, and further acetoacetate, acetone, and α-hydroxybutyrate (ketone bodies). Accordingly, three studies showed an increase in acetate or acetoacetate that reflects the onset of ketogenesis as a consequence of perturbed hepatocyte metabolism [47,79,95].

High levels of uric acid, the final oxidation product of purine metabolism, were also found in [79,84,86,88], whose increase has also been described to be an independent risk factor for liver diseases [104]. Another common observation was the presence of elevated levels of amino acids [47,74,75,76,79,84,90,91,95]. These are referred to as glutamic acid, alanine, L-glutamine, Leucine/Isoleucine, glycine, tyrosine, and L-histidine and could be attributed to increased proteolysis. Necrosis may also lead to disturbed protein synthesis and protein degradation in the liver, which would result in elevated levels of free amino acids in both the liver and blood. Amino acid concentration could therefore vary according to the liver damage degree.

Other significant metabolites in different studies were related to lipid metabolism, glycerophospholipid metabolism, and bile acid biosynthesis. Specifically, an increase in choline, which serves as a precursor molecule of phospholipids is reported in two studies [79,84]. Phospholipids are important components of the cellular membranes, and they regulate cell functions as signalling molecules. Several of the studies reviewed reported increased or decreased levels of different phospholipids, or changes in their specific structural properties [74,75,79,82,83,88,89,90,91]. It is possible that these changes might be related to the access or insertion process of the phospholipids to the membrane bilayers during hepatic regeneration. Little is known about the underlying mechanisms leading to changes in phospholipids in drug-induced hepatotoxicity, but it is possible that perturbations are related to other drug-induced events such as mitochondrial dysfunction, oxidative stress, or inflammation.

Regarding bile acid biosynthesis and homeostasis, several primary and secondary bile acids were described to be altered as a consequence of DILI processes [80,81,87,88,90,92,93]. Drug-induced damage to hepatocytes may act at different levels: modifying their biosynthesis from cholesterol, conjugation, and impairing liver uptake from blood and/or the excretion of conjugated bile acids to bile, which increases their plasma concentration. Accumulation of bile acids inside hepatocytes, because of its amphipathic nature, may induce mitochondrial disruption and toxicity with subsequent oxidative stress, and ultimately apoptosis and necrosis [105]. The large number of primary and secondary, conjugated, and unconjugated bile acids, make them useful biomarkers of DILI and other liver diseases. However, an agreement on the exact candidate bile acid species to be used as a biomarker is not fully agreed upon [106].

In another context, three studies were addressed to anticipate the susceptibility to p-cresol hepatotoxicity [43,84,90]. A hypothesis is that the individual’s capacity to sulfonate certain hepatotoxic drugs, such as APAP, and thus prevent its toxicity, can be decreased significantly by the competitive presence of p-cresol, which is also sulfonated in the liver, making such individuals more prone to undergo toxic events by other drugs. P-cresol is produced from protein-derived tyrosine by gut bacteria. Thus, individuals with urinary metabolite profiles with high bacterially originated p-cresol will be at risk because of the competitive O-sulfonation of p-cresol that reduces the capacity of hepatocytes to sulfonate and inactivate/eliminate other drugs.

**Figure 4:**
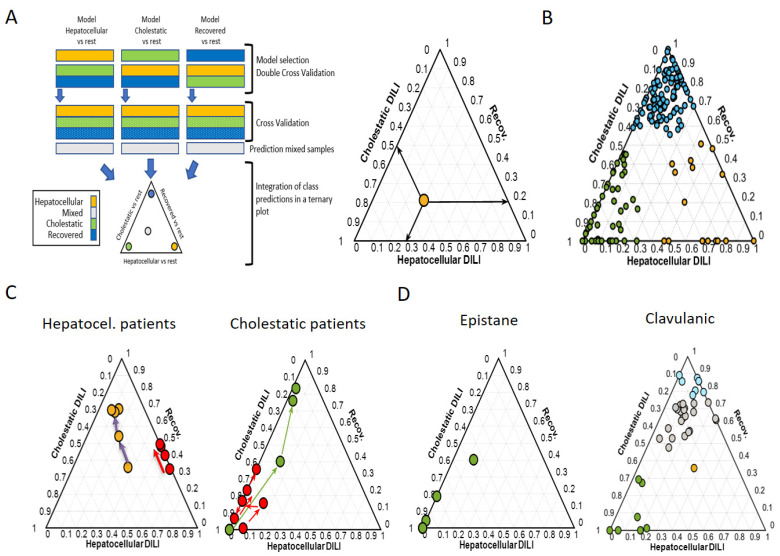
Schematic workflow of data analysis and modelling strategy to represent and interpret data in a ternary plot. (**A**) First, a PLS–DA analysis of each phenotype (hepatocellular DILI, cholestatic DILI and recovered patients) vs. the rest was carried out using the complete set of features. The set of y predicted values from the selected models were integrated into a ternary plot. A ternary plot is a two-dimensional graphical representation of three variables that sum to a constant. As PLS–DA “y” predicted values used for sample classification are unbound, “y” predicted values higher than 1 or lower than 0 were replaced by 1 or 0, respectively, and the position within the ternary plot was defined by the relative constrained “y” values. By doing this, the ternary plot was an equilateral triangle with edges to graphically depict the constrained “y”-predicted values for DILI (PLS–DA model: recovered vs. non-recovered), cholestasis (PLS–DA model: cholestasis vs. non-cholestatic), and hepatocellular (PLS–DA model: hepatocellular vs. non-cholestatic) damages. (**B**) Example of a ternary plot used to describe the different phenotypes of DILI (cholestatic, hepatocellular, and mixed, as well as those from recovered patients) predicted using the PLS–DA models. (**C**) Time-course monitoring of two patients initially diagnosed as hepatocellular and mixed-type (left triangle) or pure cholestatic (right triangle) DILI, and their evolution along the time course of the disease. While in some cases there is a clear improvement towards recovery (yellow and green dots), in others the situation worsened (red dots). (**D**) Not always that a given drug displays the same DILI phenotype in all patients. A given drug may or may not display the same DILI phenotype in all patients. While in the case of epistane all patients consistently displayed a cholestatic pattern (left triangle), in the case of clavulanic acid a wide range of mixed type DILI was observed (right panel). Dot colour represents the clinical classification at each point: green: cholestatic DILI; orange: hepatocellular DILI; gray: mixed DILI; blue: recovered patient (colour figure online). Panel A was obtained from [92].

Metabolomics has been successfully applied to accurately classify the DILI phenotype and prognosis. In a recent work, Quintás et al. [92] made use of an original approach in metabolomics to discriminate among the three DILI phenotypes (hepatocellular, cholestatic, and mixed). Upon an exhaustive search of metabolites characteristic of patients displaying the different DILI phenotypes, they found that conjugated bile acids (taurocholic acid (TCA), glycocholic acid (GCA), glycochenodeoxycholic acid (GCDCA), glycolithocholic acid (GLCA), deoxycholic acid (DCA)) and glycerophospholipids (LysoPE 18:0, LysoPE 22:6, LysoPC 16:0, LysoPC 14:0, LysoPC 18:0, LysoPC 18:1, LysoPC 17:0, LysoPC 20:3) were among the metabolite classes that discriminated the most among the cholestatic and hepatocellular DILI phenotypes. Interestingly, they used an ensemble of PLS-DA models and combined all metabolomic information into a ternary diagram that displayed, in an understandable and visual manner, the disease phenotype, the dynamic evolution of DILI progression during recovery, and the severity of the damage. These results are complementary to standard clinical biochemistry biomarkers and the R-score, providing more precise and accurate monitoring of the DILI event. For each point, the coordinates represent the probabilistic percentage of a DILI event being classified as necrosis, cholestasis, mixed type, or recovered (Figure 4A,B). Specifically, the closer to the vertices of the diagram, the more “pure” the phenotype. Thus, depending on the presence of biomarkers characteristic of necrotic DILI, cholestatic DILI, or recovery status, at each stage of the disease, the patient’s situation can be estimated along the disease time course and its eventual progression towards worsening or healing process (Figure 4C). What these innovative diagrams also allow us to examine more precisely is the DILI phenotype elicited by a given drug in different patients. Thus, there are drugs, (e.g., epistane) that consistently elicit a precise DILI phenotype in all patients, while others, (e.g., clavulanic acid) are more variable in the phenotype displayed (Figure 4D).

A further step in the in-depth data analysis of DILI metabolomics is to evaluate not the individual metabolite changes but rather their influence on the metabolic pathways they belong to. For that purpose, first, metabolites need to be properly annotated. Second, it requires the utilisation of several computational and visual tools that have been developed to identify changes in metabolic pathways, as a result of metabolite changes, such as functional analysis and pathway analysis. Functional analysis using *mummichog* starts with the putative annotation of LC-MS peaks by their mass-to-charge ratio (*m*/*z*), and the annotated features are mapped onto user-selected pathways libraries for pathway activity prediction [69]. Pathway analysis uses metabolite concentrations and pathway topological analysis to identify changes in metabolites involved in known biological pathways [107]. Output results are summarised using descriptors (*k*) as *p*-values calculated from the enrichment analysis, the total number of metabolites in the pathway, the number of metabolites present in the data set included in the pathway, or the pathway impact value, estimated as the sum of the importance measures of all metabolites in the pathway, normalised by the sum of the importance measures of all metabolites in each pathway [108].

This strategy is the one that has been used in most of the DILI research studies reviewed here and out of this, we identified several metabolic pathways consistently affected in the course of DILI in the different studies. Thus, the most frequent DILI-affected pathways were those involving amino acids, glycerophospholipids, glutathione, and bile acid metabolism, as well oxidative stress, in agreement with the known DILI pathophysiology (Figure 1). In general, despite differences at individual metabolite levels may not always be reproducible from experiment to experiment, functional and pathway analysis identifies metabolic pathways altered more consistently and reproducibly and renders more robust results regarding biological interpretability and reproducibility of DILI assessment.

**Figure 5:**
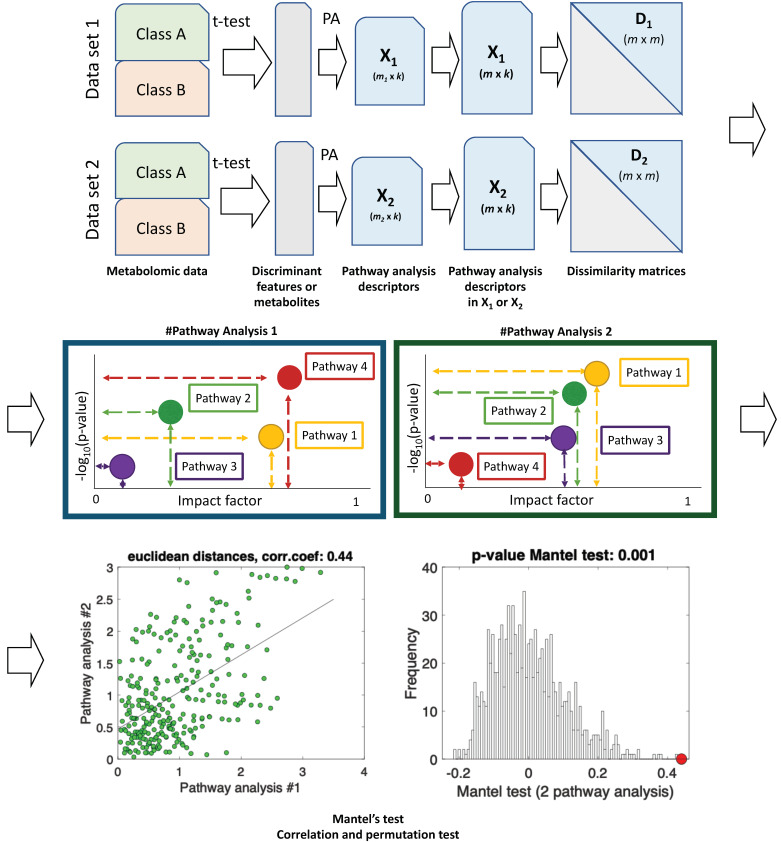
Schematic workflow for the functional correlation analysis of results from metabolic pathway analysis. Metabolomics data between two groups is compared by t-test analyses in two different datasets. Discriminant features or metabolites are identified, and metabolic pathway analysis performed. Results from pathway analysis are summarized with 2 k descriptors (the log10 (*p*-value), and the enrichment or impact factor) as coordinates of two data matrices X1 (m1 × k) and X2 (m2 × k) (m = pathways). As the number and identity of the pathways included in the results may vary across studies, for those pathways present in a single analysis, the *p*-values and the enrichment or impact factors are imputed as 1, and 0, respectively, in the other one (X1(m × k), X2(m × k). For each matrix × (m × k), a distance or dissimilarity matrix is computed (i.e., D1(m × m) and D2(m × m)) using a selected measure such as the Euclidean or the standardized Euclidean distance. Then, the lower triangular part of each dissimilarity matrix is unfolded into two vectors (d1 and d2) to calculate the pairwise linear correlation coefficient, (e.g., Pearson coefficient, or the rank-based Spearman correlation coefficient if non-linearity is expected) between them. The statistical significance of the calculated correlation coefficient between pathway analysis #1 and #2 is estimated using a permutation test and the correlation coefficient is computed for each permutation. Original figure from [73].

Third, in order to determine whether these altered metabolic pathways are specific and could constitute a characteristic metabolic fingerprint of DILI and not other liver diseases, a correlation analysis can be performed to compare the metabolic pathways altered in DILI patients and controls, with the metabolic pathways altered between patients with other liver diseases and controls. Once a correlation is estimated, a statistical test can be performed to determine the significance of this correlation. A recent work demonstrated the applicability of the Mantel’s test for the correlation of functional results from metabolic pathway analysis [73]. A schematic representation of the workflow followed in this approach is shown in Figure 5.

Its applicability and relevance as a source of information have already been validated in several studies including the analysis of reproducibility in in vitro metabolomics studies and the identification of inconsistencies between metabolomic studies outcomes [54], comparison of different phenotypes, and analysis of the degree of differentiation of hepatocyte-like cells [109], as well as in the evaluation of the toxicity mechanisms of action of certain drugs [110]. Altogether, these applications demonstrate that the algorithm is very attractive for data interpretation and meta-analysis, encouraging its use on a routine basis in clinical metabolomics studies.

## 3. Conclusions and Future Perspectives

Great advances have been made in the applicability of MS-metabolomics for the diagnosis of DILI by examining urine, plasma, and serum human samples of patients. These biological samples carry over parts of the hepatocyte exometabolome that allows the identification of DILI-specific biomarkers. These research papers support the great potential of metabolomics as a major source of novel biomarkers for DILI diagnosis and susceptibility, type and severity of the response, and effects caused by a given drug therapy. Altogether, it can contribute to a better understanding of the DILI phenomena and increase safety scrutinization of drugs under development.

Several metabolites appeared as statistically significant discriminants in those studies, demonstrating their potential as putative DILI biomarkers; however, up to today, there is not a unanimous agreement on a specific metabolic fingerprint that can be recommended for the routine diagnosis and prognosis of DILI in the clinics. Nevertheless, very promising steps forward have been undertaken and point at future clinical routine use. Inconsistencies among studies can be explained by several factors. First, the different methods applied for metabolomic analysis may influence the type and number of analytes detected and identified. Second, the majority of the studies clustered DILI and non-DILI patients without considering the DILI phenotypes and the causing agent that can belong to a wide range of pharmacological categories and, hence, influence distinct metabolic pathways. Third, the prevailing limitation for clinical verification of a novel biomarker is the requirement to test them in a meaningfully large and well-defined patient population, and here, both the number of individuals included in each cohort study and the number of studies performed so far are still insufficient to provide biomarker candidates with enough statistical discriminative power. Even more, not all studies claiming new biomarkers have proved the sensitivity and specificity of their candidate metabolites as diagnostic biomarkers of DILI. Fourth, the scope of the research differs among the studies. While some of them are focused on the identification of discriminating metabolites between DILI and non-DILI patients, others included distinct grades of DILI severity and even other types of liver diseases which difficults the identification of commonly agreed biomarkers. Therefore, there is still a need for standardised and multicentre studies to address these challenges in the field of DILI metabolomics. All this has prevented up to now, the widespread use of metabolomics-derived biomarkers in routine clinical practice. No less relevant is the fact that data analysis still requires technical expertise and highly experienced personnel. This will probably be made simpler when user-friendly analytical software for data processing will be developed and standardised.

Notwithstanding the current limitations, some studies have made very significant advances in the way DILI can be approached from the metabolome analysis, not only to discriminate the phenotype, severity, and evolution of the disease but also to bring new insights to numerically describe the behaviour of a given drug in different patients. An important outcome is that focus should be put on the comparisons of the metabolic pathways altered in DILI rather than on a combination of individual metabolites. The influences of the methodology in biassing data having been retrieved from patients in different moments and with different analysers that tend to be minimised, reaching a comparable diagnosis for a given patient’s sample. The increasing applicability of metabolomics in biomedical research has led to the development of computational and visual tools such as the *mummichog algorithm* and *pathway analysis*, as well as tools for the comparison and meta-analysis of metabolomics results, which permit extracting meaningful and more reproducible information in this type of research than with the analysis of individual metabolite levels per se.

Given the great possibilities that offer to combine physiological and metabolic pathway information, together with further technological innovation and larger cohorts of patients, the application of metabolomics will provide new findings of early specific markers of diagnostic, treatment, and prognosis of DILI.

## Supplementary Materials

The following supporting information can be downloaded at: https://www.mdpi.com/article/10.3390/metabo12060564/s1, Table S1: Studies exploring DILI biomarkers by metabolomics approaches.
