# Supplementary material for: The Potential Role of Metabolomics in Drug-Induced Liver Injury (DILI) Assessment
Source: Metabolites. 2022 Jun 19;12(6):564. doi: 10.3390/metabo12060564 (PMC9227129; doi:10.3390/metabo12060564)
Supplement: Supplementary file 1 [file metabolites-12-00564-s001.zip › metabolites-1727744-supplementary.pdf]

Study/Year/Reference	Drug	Sample	Confirmation	Other measurements	N° of patients	Platform	Up-regulated biomarkers ↑	Down-regulated biomarkers ↓	Metabolic pathways affected	Diagnostic/Predictive
Andersson et al., 2009 [74]	Ximelagatran	Plasma	ALT>3x ULN	ALT	134	LC/MS-MS, GC/MS, NMR	Putative predisposition biomarkers: L-cysteine, 36:4 PE, Creatinine, Neuraminic acid, DL-α-Aminoadipidic acid, Glutamic acid, 1-Linoleoyl-Lα-lysophosphatidic acid, Pyruvic acid, 1-Monostearinglycerol, Alanine, 2-Ketoglutaric acid. Putative treatment-effect biomarkers: 3-Hydroxy butyrate, pyruvic acid, 18:2/18:1/16:1 TG, L-glutamine, 16:1 CE, 52:5 TG, Vitamin E, 18:2/18:1/18:1 TG, 20:4/18:2/16:0 TG, 18:2/18:1/16:0 TG, 18:2/16:1/16:0 TG, 16:0/16:0 DG, L-Glutamic acid, 53:6 TG, Phenylalanine dimer, L-Phenylalanine, Phenylalanine, 53:4 TG, 18:1 LPC, 18:1/18:1/16:0 TG, 53:3 TG, 18:2/18:1/18:1 TG, Tyrosine, 18:1/18:1/18:1 TG, Tyrosine, 18:!/18:1/16:0 TG, Glutamate, 18:1/18:2 PC, Alanine 18:1/17:1/16:0 TG, Glycine-3TMS.		Pyruvate, amino acid and TG metabolism	Diagnostic and predictive. Identification of a set of putative predisposition biomarkers for ALT elevation after ximelatagran treatment and a set of putative treatment-effect biomarkers.
Clayton et al., 2009 [43]	APAP	Urine	Not confirmed	NO	99	1H NMR	*p*-cresol		Gut bacteria metabolism	Predictive. Individuals having high predoses urinary levels of p-cresol sulfate had low postdose urinary

										ratios of acetaminophen sulfate to acetaminophen glucuronide, evidencing an impaired sulfur-dependent reactive metabolite detoxification
Fannin et al., 2010 [55]	APAP	Serum	Serum ALT levels	ALT	9	NMR	Lactate		Oxidative phosphorylation	Diagnostic
Winnike et al., 2010 [47]	APAP	Urine	Serum ALT levels	ALT	71	NMR	Acetaminophen, HIS, glycine, acetaminophen-mercapturate, acetaminophen-cysteine, hydroxyl-isovaleric acid, methyl-histidine, lactate, acetate, hippurate, alanine	Creatine, creatinine, methyl-histidine, histidine, trimethylamine oxide, betaine, acetaminophen-sulfate, citrate, acetaminophen glucuronide	Glycine and glutathione metabolism	Predictive. Identification of metabolites important for discriminating responders from nonresponder after acetaminophen dosing but prior to ALT levels
Soga et al., 2011 [95]	Different drugs	Serum	Physical examination, biochemical tests, ultrasonography, CT, MRI and liver biopsies	AST, ALT, GTP, AFP, PIVKA-II	237	CE-TOF-MS	γ-Glu-Gly, γ-Glu-Ala,γ-Glu-Ser,γ-Glu-Val, γ-Glu-Taurine,γ-Glu-Leu,γ-Glu-Gln,γ-Glu-Phe, Methionine sulfoxide	γ-Glu-Citrulline unaltered	GSH metabolism and oxidative stress	Diagnostic. Identification of γ-glutamyl dipeptides as biomarkers of liver disease. γ-Glu-Citrulline is the only metabolite specific of DILI in comparison with other biomarkers that are altered in all liver diseases.

Kim et al., 2013 [77]	APAP	Urine and Plasma	Standard clinical criteria	ALT, AST, ALP, T.Bilirrubin, γ-GTP, lactate dehydrogenase (LDH).	20	1H NMR	Urine: 3-chlorotyrosine, phenylalanine and glutarate. Plasma: Lactate, glucose, 3-hydroxiisovalerate, isoleucine, acetylglycine, acetone, acetate, glutamine, ethanol and isobutyrate.	Urine: Citrate, glycine and hippurate	Dysfunction of TCAand oxidative phosphorylation and increased anaerobic glycolysis	Diagnostic.Identification of endogenous metabolites in urine and plasma samples related to APAP treatment and altered hepatic function.
Bhattacharyya et al., 2013 [78]	APAP	Plasma	Dectectable serum APAP, and dosing of APAP≥150mg/kg	APAP protein adducts, ALT, INR prothrombin time (PT)	272	UPLC-MS	Oleoyl-carnitine, palmitoyl-carnitine, myristoyl-carnitine		Acylcarnitines and β-oxidation of fatty acids	Diagnostic. Identification of increases in the long-chain acylcarnitines in children exposed to APAPin therapeutic doses or overdose. .
McGill et al., 2014 [79]	APAP	Plasma	Overdose (alt>1,000U/L and PT≥18s), Normal (ALT<100 U/L and PT<18s)	Serum APAP levels, ALT, AST, prothrombin time, bilirubin,	42	UPLC-QTOF-MS	Unable to identify in humans increased levels of acylcarnitines between groups with a large difference in liver injury, as they do identify in mice		Acylcarnitines not altered	Diagnostic. Lack of increase in acylcarnitines during APAP hepatotoxicity in humans was due in part to NAC treatment as part of their standard-of-care treatment.
Huo et al., 2014 [80]	VPA	Serum	Liver function indicator levels	ALT, AST, γ-GT, ALP. T.Bilirrubin, prealbumin.	34	UPLC–MS and 1H NMR	Phenylalanine, Uric acid, Leucine, lactate, alanine, acetate, NAC, Acetoacetate, Glutamate, Pyruvate, Choline	Creatine, LPC 16:0, PC 16:0/22:6, PC 18:2/18:2, PC 16:0/18:2, LPC 18:0, LPC 18:2, LPC 18:1, LDL/VLDL, citric acid	Glycolysis, lipid metabolism, energy metabolism,amino acid metabolism	Diagnostic. Metabonomic analysis of VPA induced hepatotoxicity in epileptic patients.

Woolbright et al., 2014 [81	APAP	Serum	Standard clinical criteria (detectable serum APAP, and/or aminotransferase level of≥1500 IU/l)	Prothrombin time, ALT, ALP, Bilirubin.	84	UPLC/QTOF-MS	TCA, GCA, GCDCA, TCDCA, TDCA, GDCA		Bile acid metabolism	Diagnostic. Suggestion that the increase in serum bile acids may be related to the degree of liver injury , which directly leads to liver dysfunction.
James et al., 2015 [82]	APAP	Serum	APAP overdose based on history ingestion and APAP levels in peripheral blood.	Serum APAP levels, APPA protein adducts, ALT .	98	UPLC-triple quadrupole MS	TDCA, GDCA,GCDCA	Taurocholic acid (TCA), Cholic acid (CA)	Bile acid metabolism	Diagnostic. Conjugate bile acids associate with toxicity severity in APAP overdose
Wang et al., 2015 [83]	Polygonum multiflorum	Plasma	International codified criteria	Heavy metals, pesticides and mycotoxins	44	LC-QTOF-MS	LysoPC(18:2), Farnesyl pyrophosphate, Oleoyl glycine, trans-Dodec-2-enoic acid, 20-Hydroxy-PGF2a, LysoPC(16:1), PC(24:0/22:0)		Lysophosphatidylcholines, phosphatidylcholines, prostaglandins and fatty acids.	Diagnostic.Differential diagnosis from other liver diseases such as AIH and HBV.
Bhattacharyya et al., 2016 [84]	APAP	Serum	Overdose: APAP> 150mg/kg and high APAP concentrations in periphe	ALT, INR prothrombin time, APAP-protein adducts	43	AbsoluteIDQ® p180 platform from Biocrates Life SciencesAG	PCs and LPCs with comparatively short chain lenghts, linked PCs, LPC-C26:1	PCs and LPCs with VLCFAs	Phospholipid metabolism	Diagnostic. Identification of significant changes in PCs and lysoPCs with VLCFAs between therapeutic, control and overdose group.

			ral blood							
Schnackenberg et al., 2017 [85]	APAP	Urine	Published guidelines	ALT	19	UPLC/QTOF-MS and NMR	2-Oxoarginine, ascobrbic acid, alanine, choline, fructose, glucose, hippurate, indoxyl, lactate, propylene glycol, pyruvate, taurocholic acid isomer, trimethyl N-oxide, uracil, uric acid	Citrulline, Cresol, Hidroxybutyrylcarnitine, Proline	Arginine and proline metabolism, TCA cycle, taurine and hypotaurine metabolism, glycine, serine and threonine metabolism, and glutathione metabolism	Diagnostic. Identification of metabolic pathways altered after APAP overdose
Lee et al., 2017 [86]	Amoxicillin/clavulanate	Urine	ALT levels	ALT, AST, ALP, T.Bilirubin	31	UPLC--QTOF-MS	7-Methylxanthine, 3-Methylxanthine, 7-Methyluric acid	Azelaic acid	Mitochondrial oxidative stress	Diagnostic. Identification of metabolomic signatures of amoxicillin/clavulanate exposure in healthy volunteers as predictors of AC-DILI.
Cao et al., 2018 [87]	Isoniazid, Rifampicin, Ethambutol, Pyrazinamide (HRZE regimen)	Urine	ALT>40 U/L	T. Bilirubin, ALT, AST	77	HPLC-LTQ-MS	Uric acid, 3-Furoic acid, 7-Cyano-7-deazaguanine, Sebacid acid, I-Methyl-4-nitro-imidazole, Mesaconic acid, cis-4-octenedioic acid, Imetit, D-Mannitol.	Hypoxanthine, 3-Hydroxy-3-methyl-Glutaric acid, Isobutyrylglycine, Pyrazineethanethiol, 2-Hydroxypropylphosphonate, p-Hydroxyphenylacetic acid, Gluconolactone, Indoxylsulfuric acid, Citramalic acid, Creatinine, L-Erythrulose, Orotinichalcone, Queuosine, N-acetylaspartate, cis-Aconitic acid, Urea, Myrigalone E, Glabrone, alpha-hydroxyisovaleric acid	Purine metabolism, arginine and proline metabolism, TCA cycle, pentose phosphate pathway.	Diagnostic. Identification of metabolites significantly increased in the DILI group compared to those in the non-DILI group after HRZE regimen.

Ma et al., 2019 [88]	Tradicional chinese medicine, Analgesic-antipyretic drugs, Antituberculosis drugs, Antibiotic drugs, Lipid-lowering drugs, Tibetan medicine, Healthcare products, Unknown medication	Serum	ALT>40 U/L and T.Bilirubin> 20µmol/L	ALT, ALP, T.Bilirubin,INR	68	UHPLC-LTQ-Orbitrap-MS	DILI vs control: Palmitic acid, TCDCA, GCA and TUDCA. Severe vs mild DILI: GCA, TCA, TUDCA, GCDCA, GCDCS, TDCA.	DILI vs control: LPE Severe vs mild DILI: CDCA, DCA and LCA.	Bile acid metabolism	Diagnostic. Identification of bile acids as biomarkers for the early diagnosis and severity of DILI
Xie et al., 2019 [89]	DILI patients	Serum	Clinical evaluation	ALT, AST, ASP, T. Bilirubin, INR, K18	90	UPLC-MS/GC-MS	Severe vs non-severe: iminodiacetic acid, glucuronic acid, GCDCA, TCDCA, biliverdin, SM(d18:1/14:0), SM(d18:0/16:1)	Severe vs non-severe: gluconic lactone, D-(glycerol 1-phosphate),uric acid, terephthalic acid, 3,4-dihydroxyphenylglycol, xanthosine, leucine, tartaric acid, N-methyl-L-glutamic acid, linoleic acid, cholesterol, maltose, sodium glycocholate, PC (22:6/16:0), PC (16:1;18:2), PC (18:0/20:3)	Primary bile acid biosynthesis, alpha-linolenic acid metabolism, glycerophospholipid metabolism, starch and sucrose metabolism	Diagnosis. Identification of metabolites related to the severity of idiosyncratic DILI
Zhang et al., 2020 [75]	Polygonum multiflorum	Serum	ALT levels. Susceptible group ALT>ULN, tolerant group ALT<1 ULN	ALT, AST, ALP, T.Bilirubin.	36	UPLC-Q-TOF-MS/MS	Glyceric acid, L-glutamate, Oxalosuccinic acid, PC 22:6, Acylglycerone phosphate, Indole-5,6-quinone, Gentisate aldehyde, Precorrin 6Y, 2E- tetradecenoyl-CoA, Ceramide, 3-Indolyllactic acid, Crotonoyl-CoA, LysoPC 20:4, Stearic acid, PE 22:6, Precorrin 8X, Kynurenic acid, 4-Hydroxyphenylacetate, Glutaric acid, Phenyllactic acid, L-Tyrosine, L-Histidinol.	Phosphoribosyl-ATP	Sphingolipid, glycerophospholipid, fatty acid, histidine and aromatic amonio acid metabolism.	Predictive. Characterization of metabolic profile of idiosyncratic DILI risk individuals before PM ingestion.

Saito et al., 2020 [90]	Suspected drugs: antibacterial agents, antineoplastic agents, anti-inflammatory and antirheumatic products, and psycholeptics.	Plasma	Clinical evaluation	AST, ALT, ALP, T.Bilirubin	54	LC-MS	Acute Mixed-type DILI vs Recovery Phase: Cer d34:1, Cer d36:1, and GM3 d34:1. Si comparamos acute phase mixed-type DILI with healthy subjects, 6 lipids were significantly different{LPC(18:2), LPC(16:1e), PE(38:6e), Cer(d34:1), Cer(d36:1)and GM+O(d34:1). Acute cholestatic-type DILI vs recovery phase: PC 30:0, PC 31:0, PC 32:1, PC 33:1.	Acute mixed -type DILI vs recovery phase: LPC 18:2, LPC16:1e, PC 38:6e, PE 36:4e, PE 38:4e, PE 38:6e. Acute cholestatic type-DILI vs recovery phase: PC 36:5e, PC 38:6e, PE36:4e, PE 38:4e, PE 40:6e, PE 40:7e, PI 38:3, PI 38:4, PI 40:4, CerG3 d40:1, CerG3 d42:1, SM d40:1, TG 44:0, and CoQ10.	Lipid metabolism	Diagnostic. Identification of lipids with a relatively high capacity to discriminate the acute phase from the recovery phase and healthy subjects. The direction of the changes of the ones compared with healthy subjects are not mentioned.
Huang et al., 2020 [91]	Polygonum multiflorum	Plasma	International codified criteria	IgG, IgM, Bilirubin, ALT, AST, ALP	58	UPLC-QTOF-MS	4-Cresol, Phenylalanine, P-cresol sulfate, Oxalosuccinic acid, D-Glucuronic acid, Deoxyribose 5-phosphate, Melatonin, Inosine, PA(17:2(9Z,12Z)/0:0)a, (25S)-5β-cholestane-3α, 7α, 12α, 26-tetrol, Glycochenodeoxycholate, Lyso PE (0:0/22:6(4Z, 7Z, 10Z, 13Z, 16Z, 19Z))b, Taurocholate, PA(14:1(9Z)/13:0)c, PG (14:1(9Z)/18:3(6Z,9Z,12Z))d, CoA, Valine, Methionine, Phenylalanine, Coniferyl aldehyde, Tyrosine, Phytosphingosine, PE (17:2(9Z, 12Z)/0:0)e, Bilirrubin, DG(17:2(9Z,12Z)/20:0/0:0)		Disturbances in some metabolic pathways, such as the metabolism of three essential amino acids (i.e., tryptophan, valine, phenylalanine), glycerophospholipid metabolism, primary bile acid biosynthesis, and sphingolipid metabolism may specifically contribute to PM-DILI.	Diagnosis. The ratios P-cresol sulfate vs phenylalanine and Inosine vs Bilirubin are the best discriminating in the differentiation of PM-DILI from HBV and AIH.

Chen et al., 2021 [92]	Herbal and traditional medicine, chemical drugs and combined used of both	Serum	DILI and liver cirrhosis guidelines	ALT, ASP, ALP, T. Bilirubin, Direct Bilirubin, Total bile acid, INR, γ-glutamyl transpeptidase, cholinesterase, total cholesterol, triglyceride, creatinine, IgA, IgG, IgM, white blood cells, platelet, albumin, globulin, albumin/globulin ratio, prealbumin	83	LC-QTOF-MS	**Cirrhosis vs non cirrhosis**: 5alpha-Androstan-3beta,17alpha-diol disulfate,2-(3-Carboxy-3-aopropyl)-L-histidine,3-Methoxyanthranilate,Taurochenodesoxycholic acid, L-Phenylalanine, Tuftsin, D-Xylulose, Citric acid, Beta-Citryl-L-glutamic acid, 9-Hydroxypalmitic acid, 3-keto stearic acid, Glycocholic acid, PC(22:6/18:3), Chenodeoxycholic acid glycine conjugate, Propionic acid, SM(d18:0/16:1(OH)), PC(14:0/20:0),3-Hydroxyanthranilic acid, (all-Z)-8,11,14-Heptadecatrienal, N(omega)-Hydroxyarginine, 5-Methyltetrahydrofolic acid, N1-Acetylspermidine, Indolepyruvate, L-Arginine, Dihydrouracil, Tryptophyl-Glutae, Cysteinyl-Proline, Enol-phenylpyruvate, Indoleacetaldehyde, 7-Methylguanine. **Decompensated vs compensated:** Isopropylmaleate, Benzoquinoneacetic acid, Quinolinic acid, 2-Hydroxyglutarate, PE(22:2/18:3), PA(20:5/22:6), Glucosylceramide (d18:1/26:1), 3-Carboxy-1-hydroxypropylthiae diphosphate, UDP-L-rhamnose,Topaquinone,2-Hydroxyphytanoyl-CoA, Selenocysteine, SM(d18:0/22:2), Bilirubin diglucuronide, Menatetrenone, Menaquinol, 3-Polyprenyl-4,5-dihydroxybenzoate.	**Decompensated vs compensated:** PS(18:0/22:6),2-hydroxy-2-(4-hydroxy-3-methoxyphenyl)acetic acid, PC(14:0/20:0), L-Proline, Trypanothione disulfide, Trihexosylceramide, 3-O-Sulfogalactosylceramide(d18:1/12:0), Phosphodimethylethanolae	Phenylalanine and tyrosine metabolism, tryptophan metabolism, arginine and proline metabolism, TCA cycle, ubiquinone and other terpenoid-quinone biosynthesis, and bile acid biosynthesis	Diagnosis. Identification of metabolic fingerprints associated with DILI cirrhosis (30 metabolites) and decompensation (25 metabolites).

Quintás et al., 2021 [93]	Different drugs	Serum	International criteria of causality. ALT and ALP levels	ALT, ALP, AST, GGT, T. Bilirubin, Albumin, Tryglycerides, Cholesterol, Glucose, Creatinine	79	UPLC-MS/MS			Bile acids, amino acids, glycerophosphocholines and steroidal glycosides	Diagnosis. Identification of a metabolic fingerprint characteristic of distinct DILI phenotypes and DILI-recovered phenotype
Xie et al., 2021 [94]	Polygonum Multiflorum, Fructus Psoraleae, Anti-tuberculosis, cephalosphorin, Azithromycin, Amoxicillin	Serum	Clinical evaluation	White blood cells, albumin, ALT, AST, total bile acid, ALP, GGT, T. Bilirubin, TG, Cholesterol, HDL, LDL, VLDL, INR	192	UPLC-MS/MS	DILI vs healthy controls: 12-ketoLCA, bUDCA, DCA, GCA, GCDCA, NorCA, TCA, TCDCA. Severe vs moderate DILI: GCDCA, TCDCA and NorCA.		Bile acids metabolism	Diagnosis.Identification of bile acids that can predict DILI and its severity
Sonn et al., 2022 [76]	APAP	Serum	ALT >60 IU/L	ALT, ALP, AST, INR, Total protein, albumin, Total and direct bilirubin, tryglicerides	204	UHPLC-MS	Diagnosis: Glycine, 5-oxoproline, 5-l-glutamyl-L-glutamine, L-citrulline higher in subjects that develop ALT elevations. Predictive biomarkers: At time 0, patients that will suffer from ALT elevation have higher levels of Ornithine, Allantoate and Nicotinamide.		Glutathione metabolism and Urea Cycle	Predictive and diagnosis. Identification of predictive biomarkers in pre-treatment samples that are predictive of subjects who will develop transient ALT elevation and subsequent hepatic adaptation.

Table S1. Studies exploring DILI biomarkers by metabolomics approaches. APAP= Acetaminophen, VPA= valproate, ALT= alanine aminotransferase, UNL= upper normal limit, CT= computed tomography, MRI= magnetic resonance imagining, PT= prothrombin time, T. Bilirubin= Total bilirrubin, ALP= Alkaline phosphatase, GTP= Guanosine Triphosphate, AFP= Alpha Fetoprotein, PIVKA-II= Protein induced by vitamin K absence-II, AST= Aspartate aminotransferase, LDH= lactate dehydrogenase , INR= International Normalized Ratio, GGT= gamma-glutamyl transferase, TG= tryglyceride, HDL= High density lipoprotein, LDL= Low density lipoprotein,

VLDL= Very low density lipoprotein, TCA= Taurocholic acid, GCA= Glycocholic acid, GCDCA= Glycochenodeoxycholic acid,, TCDCA= Taurochenodeoxycholic acid, TDCA= Taurodeoxycholic acid, GDCA= Glycodeoxycholic acid, TUDCA= Tauroursodeoxycholic acid, GCDCS= Glycochenodeoxycholic sulfate, CA= Cholic acid, 12-ketoLCA= 12-ketolitocholic acid, bUDCA= ursodeoxycholic acid, DCA= Deoxycholic acid, NorCA= Norcholic acid, CDCA= Chenodeoxycholic acid, LCA= Litocholic acid, SM= Sphingomyelin, Cer= Ceramide, GM3= monosialodihexosylganglioside, PE=Phosphatidylethanolamine, PC= Phosphatidylcholine, LysoPC= Lysophosphatidylcholine, DG= Diglycerides, VLCFAs= Very low chain fatty acids, LPE= Lysophosphatidylethanolamine, PS= Phosphatidylserine, CoQ10= Coenzyme Q10, TCA= tricarboxylic acid cycle, NAC= N-acetylcysteine, AIH= autoimmune hepatitis, HVB= hepatitis virus B, , PM= Polygonum Multiflorum.
